# Residence time of singlet oxygen in membranes

**DOI:** 10.1038/s41598-018-31901-9

**Published:** 2018-09-18

**Authors:** V. S. Sokolov, O. V. Batishchev, S. A. Akimov, T. R. Galimzyanov, A. N. Konstantinova, E. Malingriaux, Y. G. Gorbunova, D. G. Knyazev, P. Pohl

**Affiliations:** 1 A.N. Frumkin Institute of Physical Chemistry and Electrochemistry, Russian Academy of Sciences, Moscow, Russia; 2 Moscow Institute of Physics and Technology, Dolgoprudny, Russia; 3 National University of Science and Technology ‘‘MISiS’’, Moscow, Russia; 4 N.S. Kurnakov Institute of General and Inorganic Chemistry, Russian Academy of Sciences, Moscow, Russia; 5 Institute of Biophysics, Johannes Kepler University Linz, Linz, Austria

## Abstract

Photodynamic therapy uses photosensitizers (PS) to kill cancer cells by generating reactive oxygen species – like singlet oxygen (SO) - upon illumination with visible light. PS membrane anchoring augments local SO concentration, which in turn increases photodynamic efficiency. The latter may suffer from SO’s escape into the aqueous solution or premature quenching. Here we determined the time constants of SO escape and quenching by target molecules to be in the nanosecond range, the former being threefold longer. We confined PS and dipolar target molecules either to different membrane monolayers or to the same leaflet and assessed their abundance by fluorescence correlation spectroscopy or membrane surface potential measurements. The rate at which the contribution of the dipolar target molecules to membrane dipole potential vanished, served as a measure of the photo-oxidation rate. The solution of the reaction–diffusion equations did not indicate diffusional rate limitations. Nevertheless, reducing the PS-target distance increased photodynamic efficiency by preventing other SO susceptible moieties from protecting the target. Importantly, our analytical model revealed a fourfold difference between SO generation rates per molecule of the two used PSs. Such analysis of PS quantum yield in a membrane environment may help in designing better PSs.

## Introduction

Photosensitizers (PS) play a key role in cancer photodynamic therapy^1–3^. They adhere to cancer cells and kill them when excited by light due to the generation of reactive oxygen species (ROS)^4^. PS that respond to visible light may be tuned to mainly produce singlet oxygen (SO), which, in turn, preferentially targets membrane proteins. The efficacy of this approach crucially depends on (i) PS’ membrane affinity^5^, (ii) the quantum yield of SO generation, (iii) SO lifetime, *τ*_l_, and (iv) SO dwell time *τ*_dw_ in the membrane.

SO residence time *τ*_r_ may be determined by either *τ*_l_ or *τ*_dw_. Taking into account ^1^O_2_ decay^6^, SO may travel δ = √D*τ*_l_ ≈ 120 nm in first case assuming a diffusion constant of D = 5 × 10^−5^ cm^2^ s^−1^ ^7^. *τ*_l_ ≈ 3 μs has been reported for a liposomal environment^8^. Thus, once born within the membrane, the likelihood of SO hitting the desired target would appear to be rather high, even if the cell membrane is sparsely decorated by PS molecules. In the second case *τ*_r_ is limited by SO’s escape into the aqueous environment. The oxygen water/membrane distribution coefficient is equal to *K*_p_ ≈ 4.4^9^. Assuming the same *K*_p_ for SO suggests that SO may hit the membrane-water interface no more than 4.4 times before escaping into the cytoplasm or the extracellular solution. If we take membrane thickness d = 4 nm as the characteristic diffusion span between the hits, we find *τ*_dw_ = d^2^/D = 3 ns. This estimate is close to *τ*_dw_ = 12 ns as predicted by molecular dynamics simulations^10^. Accordingly, *τ*_dw_, would be much smaller than *τ*_l_, indicating that the actual distance between PS and target molecules determines photodynamic efficiency. If so, an increase in diffusion span due to PS burial into the hydrophobic membrane interior should augment *τ*_dw_ (and thus *τ*_r_), which in turn, could increase photodynamic efficiency. A correlation between PS penetration depth and photo effects has indeed been observed^11^, however, the molecular mechanism has not yet been identified.

To distinguish between the possible scenarios: *τ*_r_ ≈ *τ*_l_, or *τ*_r_ ≈ *τ*_dw_, we adsorbed PS and dipolar target (DT) molecules at different densities either to the same or to opposing leaflets of a lipid membrane and analyzed effective encounters between SO and DT in terms of the rate at which DT’s contribution to membrane dipole potential vanished. We observed *τ*_l_ to be in the nanosecond range indicating that SO rarely escapes from the membrane and that augmenting photodynamic efficiency requires shortening of the DT to PS distances.

## Materials and Methods

Black lipid membranes (BLMs) were formed by the Mueller Rudin technique^12^ from a solution containing 15 mg/ml L-α- diphytanoylphosphocholine (Avanti Polar Lipids, USA) per ml of n-decane in an aperture (diameter 0.8–1.2 mm) of a Teflon diaphragm that separated two aqueous compartments of equal volumes. Both compartments were continuously stirred by magnetic stirrers. Buffer solutions were prepared in twice distilled water with 100 mM KCl (chemically pure, Reachim, Russia) and 10 mM HEPES (Calbiochem, USA) at pH 7.5, adjusted by KOH (chemically pure, Reachim, Russia). The styryl dye 4-(2-(6-(Dibutylamino)-2-naphthalenyl)ethenyl)-1-(3-sulfopropyl)pyridinium hydroxide (di-4-ANEPPS; Sigma, USA) and aluminum phthalocyanines, AlPcSn, with various numbers *n* (1 ≤ *n* ≤ 4) of peripheral sulfo-groups from (Porphyrin Products, USA) served as DT and PS, respectively.

The electrical measurements were performed with the aid of silver-chloride electrodes that were connected to the aqueous compartments via agar bridges. Total electrode resistance did not exceed 30 kOhm. Membrane capacitance and conductance were continuously measured as previously described^13^. The difference of BLM boundary potentials, *Δφ*_*b*_, was monitored by using the intramembrane field compensation (IFC) method^13,14^ (see also reviews^15,16^). IFC uses a variable dc offset to a sine wave input (300–700 Hz) to minimize membrane capacitance. We measured *Δφ*_*b*_ and determined the photo–oxidation rate of DT as previously described^17^. In brief, we placed the planar lipid bilayer into the focused beam of a monochromatic light source (semiconductor diode laser with a wavelength of 670 nm, optical power 1 mW). Subsequently, we added the PS into the distant aqueous compartment (also called *cis* compartment) with respect to the light source. DT was added either to the *cis* compartment or to the opposite (*trans*) compartment. Since both substances are membrane impermeable, their location remained well defined throughout the experiment”.

The membrane surface densities, *T* and *P*, as well as the lateral diffusion coefficients, *D*_*DT*_ and *D*_*PS*_ of both DT and PS were assessed by placing the horizontal BLM formed by Montal-Mueller technique^18^ into the focus of a laser scanning microscope (LSM 510 META/ConfoCor 3, Carl Zeiss, Jena, Germany) and exploiting fluorescence correlation spectroscopy, FCS^19,20^:

1 $${D}_{DT,PS}=\frac{{r}^{2}}{4{\tau }_{d}},$$

where *r* and *τ*_d_ are the lateral focus radius of the confocal volume and the characteristic residence time of DT or PS in the confocal volume, respectively. *τ*_d_ was obtained by fitting a one-component model for 2D translational diffusion^21^ to the autocorrelation function *G* of the time, *τ*, dependent fluorescence intensity:

2 $$G(\tau )=1+\frac{1}{N(1+\tau /{\tau }_{D})},$$

where *N* is the average number of fluorescent molecules in the confocal volume.

## Results

### PS adsorption to the lipid bilayer

**Figure 1:**
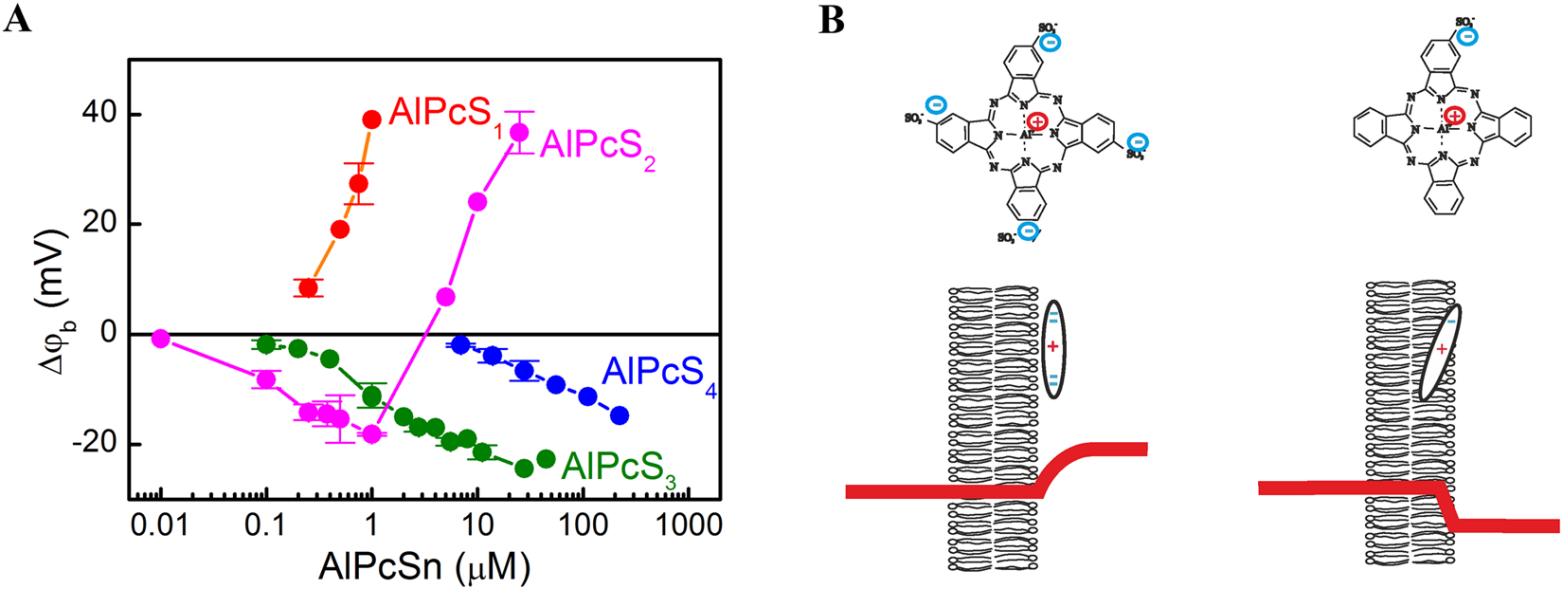
Adsorption of phthalocyanines on the bilayer lipid membrane. (**A**) Dependence of the boundary potential change Δφ_b_ upon concentration of aluminum phthalocyanines with various numbers of sulfo groups in the solution. (**B**) The various positions of the phthalocyanines in the membrane with four and one sulfo groups resulting in the generation of the boundary potential of opposite signs on the surface of the lipid membrane. Red lines – the profiles of the potential change across the membrane due to adsorption of these phthalocyanines on the right side of the membrane.

PS membrane adsorption alters bilayer boundary potential *φ*_*b*_. The introduced change Δ*φ*_*b*_ depends upon PS’ aqueous concentration, *P*_a_^5,13^ (Fig. 1A). Generally, *Δφ*_*b*_ is a superposition of changes in membrane surface potential *Δφ*_s_ and membrane dipole potential *Δφ*_d_^15,16^:

3 $$\Delta {\phi }_{b}=\Delta {\phi }_{{\rm{s}}}+\Delta {\phi }_{{\rm{d}}}$$

*φ*_s_ is accessible via measurements of the electrophoretic mobility of lipid vesicles. The mobility reflects the so-called ζ – potential that, in 0.1 M KCl, describes the electrostatic potential at 0.2 nm distance from the vesicle surface. Using the Gouy Chapman theory, *φ*_s_ can be calculated from ζ. Upon AlPcS_3_ and AlPcS_4_ membrane adsorption, we measured *Δφ*_*b*_ values that can entirely be attributed to changes in ζ^5^, i.e. *Δφ*_*b*_ and *Δφ*_s_ are roughly identical.

In contrast, for AlPcS_1_ and AlPcS_2_ negative ζ – values^5^ (and thus negative *Δφ*_s_ values) have been observed for all aqueous PS concentrations *P*_a_, while *Δφ*_*b*_ was positive for some AlPcS_2_ concentrations and all AlPcS_1_ concentrations. The observation suggests that both AlPcS_2_ and AlPcS_1_ significantly alter *Δφ*_d_. The underlying dipole moment of the phthalocyanine molecule is generated by the separation of the positive charge at the aluminum cation in the center and the negative charges of the sulfo-groups on its periphery. Thus, in contrast to AlPcS_4_, the charges in AlPcS_1_ are distributed asymmetrically (Fig. 1B). The positive sign of *Δφ*_d_ indicates that AlPcS_1_ inserts the positive Al-moiety into the lipid bilayer, whereas the negatively charged S-group faces the aqueous solution (Fig. 1B). AlPcS_2_ is likely to adopt a similar orientation (Fig. 1B). Our result is in line with AlPcS_n_ accessibility by fluorides^5^ and molecular dynamic simulations with porphyrin^22^.

### Determination of membrane surface PS and DT densities

**Figure 2:**
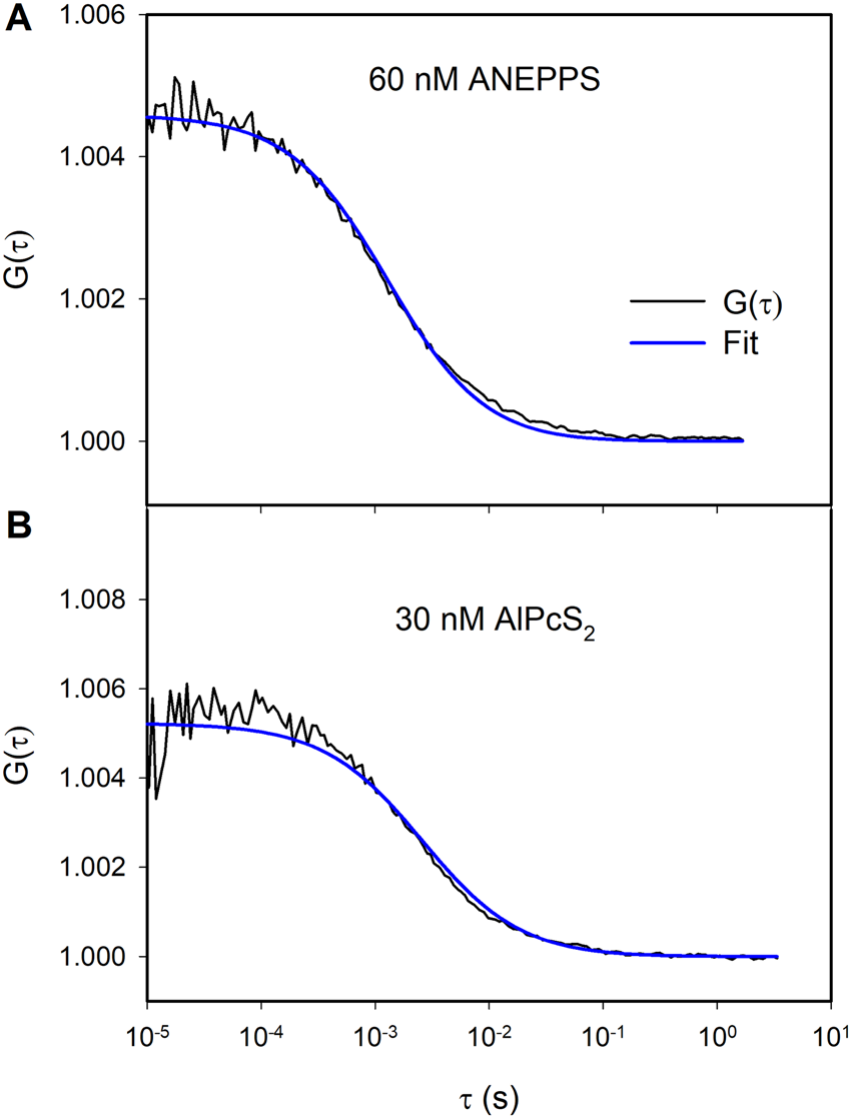
(**A**) Autocorrelation function of fluorescence of di-4-ANEPPS (concentration in solution is 60 nM) and its approximation by the equation (2). (**B**) Autocorrelation function of fluorescence of AlPcS_2_ (concentration in solution is 30 nM) and its approximation by the equation (2). BLM formed by the Montal-Mueller technique by adding the solution of diphytanoylphosphatidylcholine in hexane (15 mg/ml) to the water-air interface.

We performed FCS experiments to find *T* and *P* (Fig. 2). DT elicits *Δφ*_*b*_ changes that are proportional to its aqueous concentrations^17^. That is, DT’s limited aqueous solubility prevents the linear relation between *T*_a_ and *T* from breaking down as might be expected in case of a Langmuir adsorption isotherm. Since *Δφ*_*b*_ and the aqueous DT concentration *T*_a_ are the proportional to each other^17^, there must be a linear relationship between *T*_a_, and *T*. It allowed calculating that *T* amounts to 30 molecules per μm^2^ and per nM of *T*_a_. Thus, for *T*_a_ = 60 nM we find *T* = 1800 molecules μm^−2^ (Fig. 2A). Accordingly, DT’s contribution to *Δφ*_*b*_ amounts to 0.003 molecules/nm^2^/mV.

From ^*τ*^*DT* = 1.31 ms, we found *D*_*DT*_ = 7.6 µm^2^/s (Eq. 2). We used FITC (fluorescein isothiocyanate) for calibration experiments. Its diffusion coefficient *D*_FITC_ is equal to 565 µm^2^/s^23,24^. From the equation:

4 $$V={\pi }^{\tfrac{3}{2}}{({\omega }_{r}^{2})}^{\tfrac{3}{2}}S={\pi }^{\tfrac{3}{2}}{(4{D}_{FITC}{\tau }_{FITC})}^{\tfrac{3}{2}}S$$

we determined the confocal volume *V* = 0.22 fl. The structural parameter *S* (defined as the ratio of the long (ω_z_) to the short radii (ω_x_ = ω_y_ = ω_r_) of the ellipsoidal confocal volume and *τ*_*FITC*_ were equal to 5 and 18 µs, respectively.

Similarly, we obtained *D*_*PS*_ = 3.4 µm^2^/s from PS residence time in the focal plane *τ*_*PS*_ = 2.6 ms. At an aqueous AlPcS_2_ concentration *P*_a_ = 30 nM (Fig. 2B), we observed *P* = 1700 molecules/μm^2^. The corresponding adsorption coefficient amounts to 57 molecules/μm^2^ per nM *P*_a_. The focal volume of the red laser was calibrated with Cy5. We took Cy5’s diffusion coefficient and *S* to be equal to 280 µm^2^/s^25,26^ and 8, respectively. Cy5’s residence time ^*τ*^_*Cy*5_ amounted to 31.6 µs.

### The photodynamic activity of phthalocyanines with various numbers of sulfo-groups

**Figure 3:**
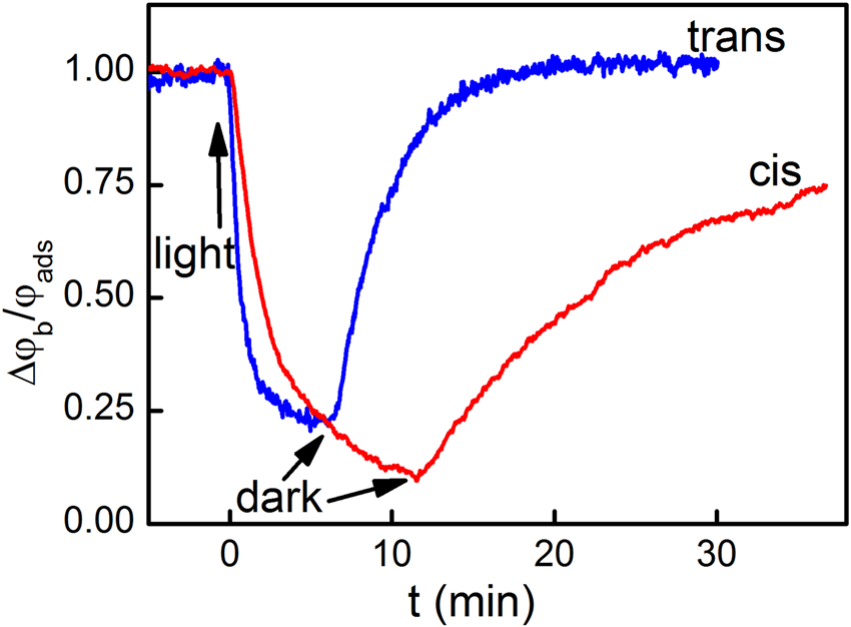
Comparison of the kinetics of relative potential decay during illumination and its recovery in the dark in case of cis and trans photo effects. Either the *cis* or the *trans* solutions contained 2 μM di-4-ANEPPS (“cis” photo effect or “trans” photo effect, respectively). 0.2 μM AlPcS_2_ were added to the cis solution.

**Figure 4:**
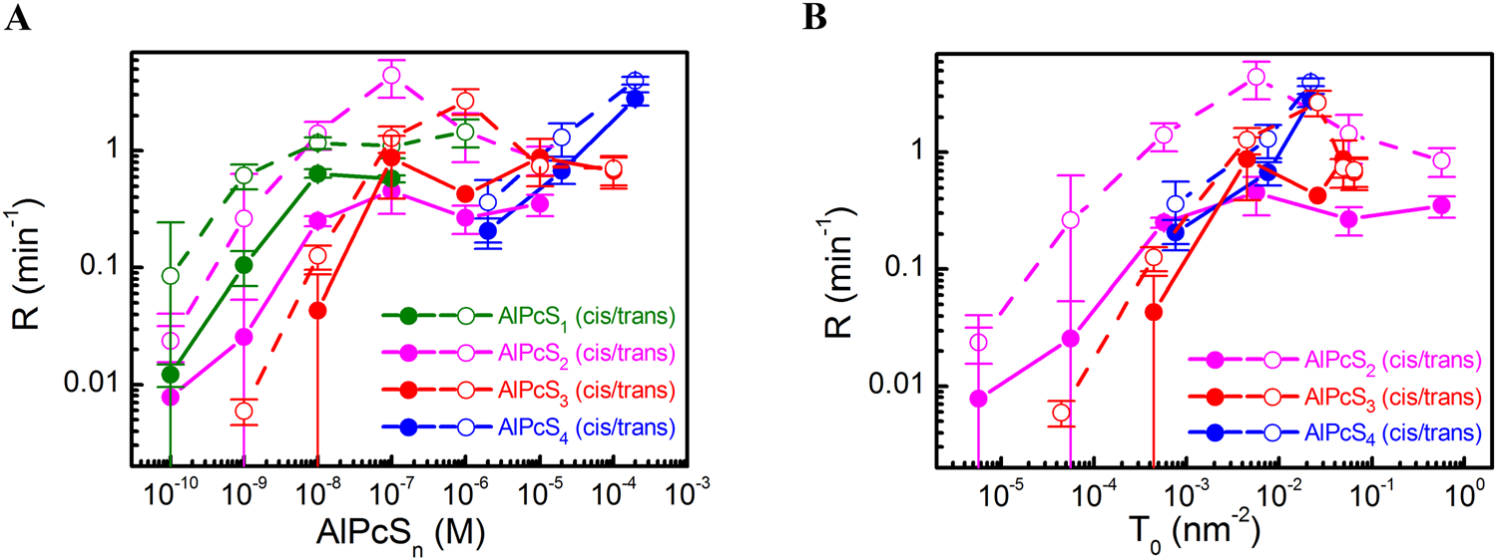
Dependence of the rate *R* of oxidation of di-4-ANEPPS adsorbed on the *cis* (open symbols) or *trans* (closed symbols) side of the BLM on the aqueous AlPcS_n_ concentration (**A**) or as a function of AlPcS_2_ and AlPcS_4_ membrane surface densities (**B**).

When both DT and PS were adsorbed to the BLM surface, membrane illumination at 670 nm led to a drop in *Δφ*_*b*_ due to DT’s oxidation by SO^17^. *Δφ*_*b*_ recovered in the dark due to the adsorption of intact DT molecules from the aqueous bulk solution (Fig. 3). The kinetics of photo damage depended on whether DT and PS were added into the solution to the same (*cis* configuration) side or to the opposite (*trans* configuration) side of the membrane. Dividing *Δφ*_*b*_(*t*) by its value *φ*_ads_ at time *t* = 0 we define the rate *R* of DT oxidation as^17^:

5 $$R=\frac{1}{{\varphi }_{ads}}{\frac{d\varphi (t)}{dt}|}_{t=0},$$

enabling a quantitative analysis of the photodynamic effects. We found that *R* depended (i) on the number of sulfo-groups per PS molecule (Fig. 4A), (ii) the geometrical arrangement (*cis* or *trans* configuration), and (iii) the aqueous PS concentration. For PS molecules with two sulfo-groups, *R* depended non-monotonically on *P*_a_ in *trans* configuration. Similar bell-shaped concentration dependencies were reported for SO-mediated gramicidin inactivation^5^. In contrast, *R* increased monotonically with *P*_a_ in *cis* configuration, suggesting that SO quenching by PS may be involved. This would be in line with chemoluminescence-based observations of SO quenching by PS^27^.

In order to account for differences in AlPcS_n_ membrane affinities, we replotted *R* as a function of *P* (Fig. 4B). For AlPcS_3_ and AlPcS_4_, we calculated *P* from the increment in surface charge^5^, whereas we used FCS to determine *P* of the weakly charged AlPcS_2_ and AlPcS_1_. *R* did not significantly vary with the number of sulfogroups in *ci*s configuration: *R* was roughly proportional to *P* for *P* < 0.01 molecules/nm^2^, and it approached saturation at higher *P*. However, in *trans* configuration, we observed a 10-fold augmented *R* for AlPcS_2_ and the absence of saturation for AlPcS_4_. The latter may be due to AlPcS_4_’s low binding constant.

### Mathematical model

Our counterintuitive observation that R is smaller if PS and DT are adsorbed to the same leaflet than to opposite ones has three possible explanations: (1) PS and DT molecules interact with each other thereby decreasing the quantum yield of SO, (2) PS molecules quench SO or (3) DT molecules possess two different moieties that may serve as a SO target: damage of only one of these moieties - of the aniline group - changes membrane dipole potential, whereas targeting the second moiety - the unsaturated hydrocarbon chain in the middle of the molecule - remains electrically silent.

**Figure 5:**
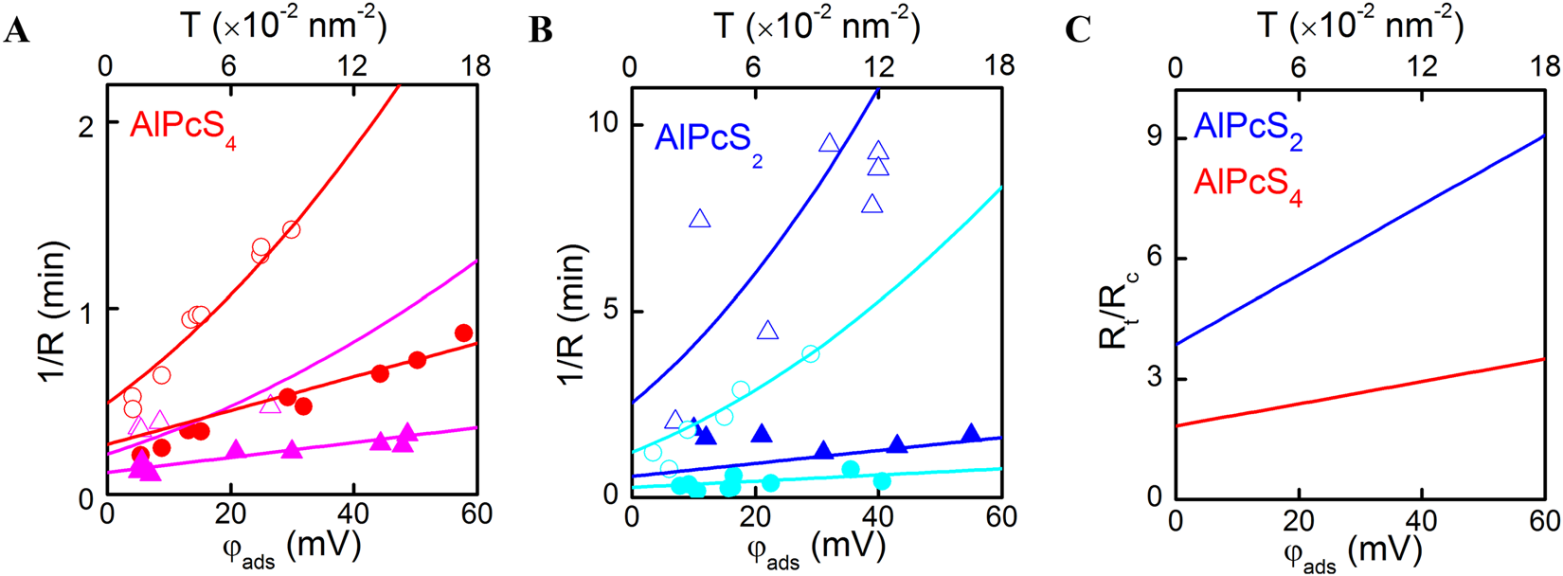
Dependence of the inverse rate of cis and trans photo effects on the potential caused by adsorption of di-4-ANEPPS at (**A**) 20 μM (squares) or 200 μM of AlPcS_4_ (down triangles) and (**B**) 4 nM (up triangles) or 200 nM (circles) of AlPcS_2_ in water solution. The filled symbols represent the experiments where di-4-ANEPPS was at *trans* side of the BLM, the open ones – at the *cis* side. The spline lines are best fits of the theoretical model (equations T4 and T5) to the data. (**C**) R_t_/R_c_ = f(*T*_0_) plot from the results of the fit.

First, we ruled out the potential interaction by recording the effect of DT on the fluorescence spectra of liposomal PS (see Supplementary Fig. S1). Since there was none, a DT-PS interaction is unlikely. Second, we estimated the impact of PS mediated SO quenching. Since it should depend on the concentration of both molecules, we would expect the ratio R_c_/R_t_ to vary with PS concentration. The invariance of that ratio at low PS concentrations (Fig. 4) excludes SO quenching by PS from being responsible for the difference between R_c_ and R_t_. Third, to estimate the impact of SO quenching by DT molecules, we plotted 1/R against *φ*_ads_ (Fig. 5). The slopes of the curves in “*cis*” configuration exceeded the slopes in “*trans*” configuration for all *φ*_ads_, thereby confirming DT’s quenching effect. The difference between the two configurations was more pronounced when AlPcS_4_ was substituted for AlPcS_2_, suggesting a deeper bilayer penetration depth of AlPcS_2_, and thus, a smaller PS to DT distance.

**Figure 6:**
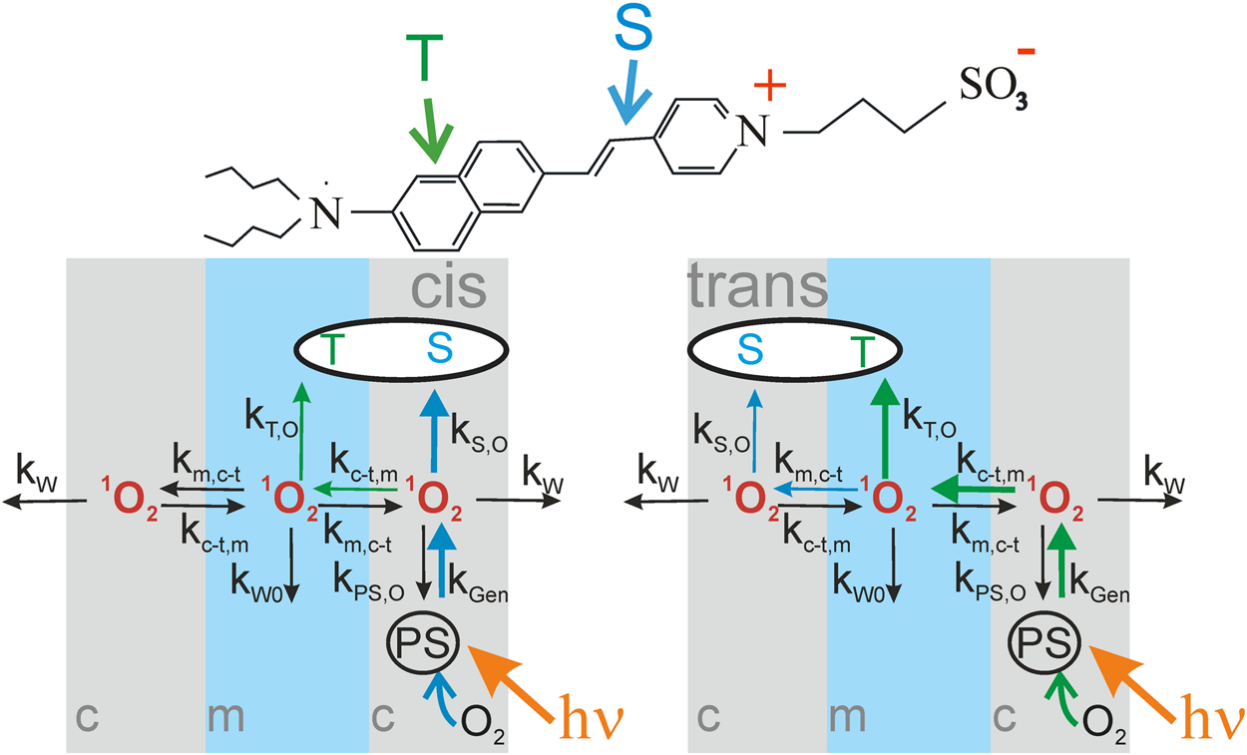
Model of SO generation, quenching and transport. (**A**) ANEPPS’ structure reveals two different moieties that can be targeted by SO: the aniline ring (T) and the unsaturated hydrocarbon chain (S). (**B**) SO generation, membrane transport, and quenching. After oxidation of the aniline ring, the target molecule loses its dipole moment (or its orientation), but the hydrocarbon double bond may still quench SO.

Differences in the oxidation rates of styryl dyes analogues^17^ indicate that DT possess two different moieties that may serve as a SO target: damage of only one of these moieties - of the aniline group - changes membrane dipole potential, whereas targeting the second moiety - the unsaturated hydrocarbon chain in the middle of the molecule - remains electrically silent. This peculiarity generates the different *R* in *cis* and *trans* configurations: In *cis* configuration, DT’s naphthalene ring is only ~ 1 nm farther away from the PS molecule than DT’s double bond (Fig. 6). Consequently, the double bond may provide protection for the naphthalene ring. In *trans* configuration, the naphthalene ring is closer to the PS molecule, and thus, it may represent the primary SO target. This hypothesis only makes sense if *τ*_r_ is very short – small enough to prevent SO from reaching distant targets, and definitely much shorter than suggested by the previously estimated *τ*_l_ = 3 µs in a liposomal suspension.

We supported the hypothesis about 2 SO susceptible DT moieties with a mathematical model that takes into account all chemical reactions and membrane diffusion processes (see Theory). The numerical solution of the differential equations satisfactorily fits the experimental data (Fig. 5) for the parameter set displayed in Table 1. The model divides the lipid membrane into three layers and assumes that SO is generated by PS molecules in one of the outer layers. In contrast, DT molecules extend from the outer to the middle layer. The Δ*φ*_d_ affecting moiety (designated by letter T in Fig. 6) - the naphthalene group at the end of the chromophore molecule - is immersed into the middle layer^17^. The other moiety that is susceptible to SO – the unsaturated hydrocarbon chain in the middle of the molecule (designated by letter S in the Fig. 6) – localizes to one of the outer membrane layers. SO may quench the S-group even when the T-moiety is oxidized and DT’s contribution to Δ*φ*_d_ has vanished.

**Table 1:** Model parameters.

Phthalocyanine	AlPcS_4_	AlPcS_2_
*k*_*Gen*_ (*m*^2^*s*^−1^)	0.6 ± 0.1	2.4 ± 0.3
***k***_***W***_ (***s***^**−*1***^)	(5.0 ± 0.9) · 10^7^	
***k***_***W0***_ (***s***^**−*1***^)	(2.9 ± 0.3) · 10^7^	
***k***_***T***,***O***_ (***m***^**2**^***s***^**−1**^)	**>10** ^**−10**^	
*k*_*S*,*O*_ (*m*^*2*^*s*^−1^)	(1.5 ± 0.2) · 10^−10^	(6.0 ± 0.7) · 10^−10^
*k*_*c*−*t*,*m*_ (*s*^−1^)^***fixed***, ***from Pm***^	2.4 · 10^8^	
*k*_*m*,*c*−*t*_ (*s*^−*1*^)^***fixed***, ***from Pm***^	5.5 · 10^7^	

They represent the best fit of the system of differential equations (T4) to the experimental data (Fig. 5A,B). The global parameters (independent on the choice of the phtalocyanine) are highlighted in bold. The local parameters, i.e. the rate, *k*_*Gen*_, of SO generation and the rate, *k*_*S*,*O*_, of SO quenching by S, are valid for all concentrations of the specific phtalocyanine.

We noticed that - contrary to the expectations - the limit of the oxidation rate at zero DT density depends on which side of the membrane contains DT (Fig. 5). This observation suggests that the DT preparation already contained oxidized molecules, which were capable of capturing SO, while being unable to contribute to *Δφ*_*b*_.

## Theory

### Mathematical model of SO generation, membrane permeation, and quenching

The model describes the permeation of singlet oxygen through the membrane taking into account the non-uniform distribution of SO across the membrane. SO mirrors the bell–shaped distribution of ground-state oxygen with the concentration maximum in the middle of the membrane^10^. The steady-state concentration of SO not only depends on this distribution, but also on its lifetime. To simplify this distribution, we will consider it to be discrete. Let the membrane consist of three layers, two of them are the fields of the lipid bilayer lying near its interface with water, and the third one – the internal hydrophobic region of the membrane, is located in the layers of hydrocarbon chains of the lipids. We will refer to these layers as *cis* (the layer with the PS molecules), medium (the middle of the membrane) and *trans* (the layer opposite to cis). The concentrations of singlet oxygen in these layers will be designated as *O*_*c*_, *O*_*m*_ and *O*_*t*_, correspondingly. The singlet oxygen is generated from the excited molecules of PS adsorbed on the cis-side of membrane. No matter which of the locations of the PS molecules is considered, ground state oxygen is present at saturating concentrations with respect to SO generation. Consequently, both AlPcS_2_ and AlPcS_4_ operate at a rate that is solely governed by light intensity (see for example Fig. 5 in^13^ or Fig. 8 in^17^).

The target of SO can be located either in the cis or trans layer. The model assumes that two parts of the target molecule react with singlet oxygen (Fig. 6). The first one, the aniline group is designated by “T”. The oxidation of this group by singlet oxygen in the middle layer (*O*_*m*_ in Fig. 6) nullifies their contribution to membrane dipole potential. The concentrations of this group in cis and trans layers are correspondingly designated, as *T*_*c*_ and *T*_*t*_. The other part – the unsaturated hydrocarbon chain in the middle of the molecule (designated by “S” in Fig. 6) can react with singlet oxygen in the surface layers (*O*_*c*_, or *O*_*t*_) without changing the dipole potential. The approach neglects the incremental diffusion span of no more than 0.5 nm that a SO molecule has to cross if born on the “wrong” (opposite to the direction of movement) side of a PS molecule. It corresponds to travel time of only ~ 1 ns, which is at least 10 times smaller than the most conservative estimate of *τ*_dw_ = 12 ns from molecular dynamics simulations^10^.

The reactions and transfer of singlet oxygen in *cis* and *trans* photoeffects are shown in Fig. 6. The singlet oxygen can react with target molecules, be quenched by the medium, the molecules or photosensitizer and transfer to the adjacent layer. Once SO has left the membrane, the probability of its retrieval is negligible since (i) the volume of the aqueous bulk so much larger than that of the membrane, (ii) and the lifetime in aqueous solutions is very limited. The equations describe the damage of the target and the processes with singlet oxygen in the experiment, where the target molecules located at the *cis* side of the membrane are

T1 $$\{\begin{array}{c}\frac{d{T}_{c}(t)}{dt}={k}_{Sol,Mem}{T}_{S}-{k}_{Mem,Sol}{T}_{c}(t)-{k}_{T,O}{T}_{c}(t){O}_{m}(t),\\ \frac{d{O}_{c}(t)}{dt}={k}_{Gen}PS-{k}_{S,O}{S}_{c}(t){O}_{c}(t)-({k}_{PS,O}PS+{k}_{c-t,m}+{k}_{W}){O}_{c}(t)+{k}_{m,c-t}{O}_{m}(t),\\ \frac{d{O}_{m}(t)}{dt}={k}_{c-t,m}({O}_{c}(t)+{O}_{t}(t))-(2{k}_{m,c-t}+{k}_{W0}){O}_{m}(t)-{k}_{T,O}{T}_{c}(t){O}_{m}(t),\\ \frac{d{O}_{t}(t)}{dt}={k}_{m,c-t}{O}_{m}(t)-({k}_{c-t,m}+{k}_{W}){O}_{t}(t),\end{array}$$

where

*k*_*a*_ — rate constant of adsorption of target molecules from solution to membrane;

*k*_*d*_ — rate constant of desorption of target molecules from membrane to the solution;

*k*_*T*,*O*_ — rate constant of damaging of target molecules by singlet oxygen;

*k*_*Gen*_ — rate constant of generation of singlet oxygen;

*k*_*S*,*O*_ — rate constant of quenching of singlet oxygen by the target molecules;

*k*_*PS*,*O*_ — rate constant of quenching of singlet oxygen by the photosensitizer molecules;

*k*_*c*-*t*,*m*_ — rate constant of transfer of singlet oxygen from cis or trans layers to the middle one;

*k*_*m*,*c*-*t*_ — rate constant of transfer of singlet oxygen from the middle layer to cis or trans ones;

*k*_*W*_ — rate constant of quenching of singlet oxygen in cis and trans layers;

*k*_*W*0_ — rate constant of quenching of singlet oxygen in the middle layer.

Similarly, the equations describing the damage of the target at the *trans* side are

T2 $$\{\begin{array}{c}\frac{d{T}_{t}(t)}{dt}={k}_{Sol,Mem}{T}_{S}-{k}_{Mem,Sol}{T}_{t}(t)-{k}_{T,O}{T}_{t}(t){O}_{m}(t),\\ \frac{d{O}_{c}(t)}{dt}={k}_{Gen}PS-({k}_{PS,O}PS+{k}_{c-t,m}+{k}_{W}){O}_{c}(t)+{k}_{m,c-t}{O}_{m}(t),\\ \frac{d{O}_{m}(t)}{dt}={k}_{c-t,m}({O}_{c}(t)+{O}_{t}(t))-(2{k}_{m,c-t}+{k}_{W0}){O}_{m}(t)-{k}_{T,O}{T}_{t}(t){O}_{m}(t),\\ \frac{d{O}_{t}(t)}{dt}=-\,{k}_{S,O}{S}_{t}(t){O}_{t}(t)+{k}_{m,c-t}{O}_{m}(t)-({k}_{c-t,m}+{k}_{W}){O}_{t}(t).\end{array}$$

To simplify the solution of these equations, we will consider that the processes described here are fast when compared with the processes of oxidation and diffusion of the targets described in^17^. If the concentration of the singlet oxygen changes much faster than that of the targets, it reaches the steady-state value before the concentration of the target significantly begins to change. Therefore, we can solve the equations assuming the steady-state distribution of singlet oxygen and equilibrium distribution of the targets.

T3 $$\frac{d{O}_{c}(t)}{dt}=\frac{d{O}_{m}(t)}{dt}=\frac{d{O}_{t}(t)}{dt}=0$$

In this case, the equations for the singlet oxygen concentration transform to the algebraic ones. By solving these equations and introducing the steady-state concentrations of the singlet oxygen into the first equations in (T1) and (T2) we only have single differential equations describing the kinetics of oxidation of the target either in cis or in trans positions. Thus we will instead solve these equations by determining the rate of oxidation of the target *R*_*c*_ and *R*_*t*_ at cis and trans positions, respectively, as a slope (time derivative) of the relative change of the potential at the beginning of illumination according to Eq. 5. The relation between the potential measured in the experiment and the surface density of the target molecules can be established experimentally by comparing the dipole potentials measured by IFC method at given concentration of the ANEPPS in the solution (the data in^17^) and the surface density of these molecules measured by the FCS technique (Fig. 2). These values are proportional to each other:

$$\phi =\alpha {T}_{c},\,{\rm{or}}\,\phi =\alpha {T}_{t},\,{\rm{where}}\,\alpha =4.65\cdot {10}^{-19}{\rm{V}}\cdot {{\rm{m}}}^{2}.$$

Relative potential can be derived through the ratio of current surface density of the target to the initial one corresponding to equilibrium of targets between the membrane and solution before the illumination

$${\phi }_{{\rm{Re}}l}(t)=\frac{\phi (t)}{{\phi }_{ads}}=\frac{{T}_{c}(t)}{{T}_{0}}=\frac{{T}_{t}(t)}{{T}_{0}},$$

where

$${T}_{0}={T}_{c0}={T}_{t0}={T}_{s}\frac{{k}_{Sol,Mem}}{{k}_{Mem,Sol}}.$$

In these designations, the *R*_*c*_ can be derived through the steady-state concentrations of singlet oxygen *O*_*m*_ determined by equations (T1)

$${R}_{c}=-\,{\frac{d{\varphi }_{{\rm{Re}}l}(t)}{dt}|}_{t=0}=-\,\frac{1}{{T}_{0}}{\frac{d{T}_{c}(t)}{dt}|}_{t=0}={k}_{T,O}{O}_{m},$$

and similarly *R*_*t*_ – through *O*_*m*_ determined by equations (T2)

$${R}_{t}=-\,{\frac{d{\varphi }_{{\rm{Re}}l}(t)}{dt}|}_{t=0}=-\,\frac{1}{{T}_{0}}{\frac{d{T}_{t}(t)}{dt}|}_{t=0}={k}_{T,O}{O}_{m}$$

For further simplification of equations (T1 and T2), we will neglect SO quenching by PS, putting the corresponding constant, *k*_*PS*,*O*_, equal to 0. It may be valid if we use very low concentrations of photosensitizers in the linear region of the dependence of the rate R on the concentrations of photosensitizers in the solution or on their surface densities (Fig. 4). It seems natural to assume that the surface densities of two parts of the same targets molecule, T and S, are equal. However, one cannot exclude the existence of a fraction of the target molecules, which are partially damaged and do not contribute to the dipole potential, but still can react with singlet oxygen and quench it. To take into account these “invisible” target molecules we will assume that surface density at each side of the membrane consists of two members: “visible” T and “invisible” S_0_

$${{\rm{S}}}_{{\rm{c}}}={{\rm{T}}}_{{\rm{c}}}+{{\rm{S}}}_{0},\,{{\rm{S}}}_{{\rm{t}}}={{\rm{T}}}_{{\rm{t}}}+{{\rm{S}}}_{0}$$

Solving the equations (T1 and T2) with the above-mentioned simplifications gives the rates *R*_*c*_ and *R*_*t*_ as functions of the ANEPPS adsorption potential

T4 $$\begin{array}{c}\frac{1}{{R}_{c}}={a}_{1}{\varphi }^{2}+{a}_{2}\varphi +{a}_{3}\\ \frac{1}{{R}_{t}}=\frac{1}{{R}_{c}}\frac{{b}_{1}}{{b}_{2}+\alpha {S}_{0}}\end{array}$$

with

$$\begin{array}{c}{a}_{1}=\frac{{k}_{S,O}}{{k}_{c-t,m}\cdot {k}_{Gen}\cdot PS\cdot {\alpha }^{2}},s\cdot {V}^{-2}\\ {b}_{1}=\alpha \frac{{k}_{c-t,m}+{k}_{W}}{{k}_{S,O}},V\\ {b}_{2}={b}_{1}+{\phi }_{0},V\\ \begin{array}{lcc}{a}_{2} & = & \frac{{k}_{S,O}}{{k}_{c-t,m}\cdot {k}_{Gen}\cdot PS\cdot \alpha \cdot {k}_{T,O}}(\frac{2{\phi }_{0}{k}_{T,O}}{\alpha }+\frac{{k}_{T,O}({k}_{c-t,m}+{k}_{W})}{{k}_{S,O}}+{k}_{W0}+{k}_{m,c-t}\\  &  & +\frac{{k}_{m,c-t}\cdot {k}_{W}}{{k}_{c-t,m}+{k}_{W}}),\,s\cdot {V}^{-1}\end{array}\\ {a}_{3}={a}_{2}{\phi }_{0}-{a}_{1}{\phi }_{0}^{2}+\frac{2{k}_{m,c-t}{k}_{W}+({k}_{c-t,m}+{k}_{W}){k}_{W0}}{{k}_{c-t,m}{k}_{Gen}\cdot PS\cdot {k}_{T,O}},s\end{array}$$

We used equation T4 to fit the data in Fig. 5. *k*_*c*-*t*,*m*_ was not used as a fitting parameter. but was obtained from the known membrane permeability *P*_M_ = 80 cm/s of O_2_^28,29^ that we assume to be equal to P_M_ of SO: ${P}_{M}={K}_{p}\frac{{D}_{M}}{\delta }$, where *δ* is the membrane thickness. Substituting SO’s membrane diffusion coefficient *D*_M_ by the Einstein relation, we find ${P}_{M}={K}_{p}\frac{{\delta }^{2}}{2\tau \delta }={K}_{p}\frac{\delta }{2\tau }\,$. Since we are not interested in the time *τ* that SO takes to cross the bilayer, but in the time that SO requires to diffuse from the middle of one of the outer layers to the middle of the membrane, we substitute *δ* and 1/*τ* for *δ*/3 and *k*_*c*-*t*,*m*_, respectively. This yields *k*_*c*−*t*,*m*_ = 2.4 × 10^8^ s^−1^.

S_0_ was treated as a constant parameter for each of PS and was found from the global fit as *αS*_0_ = 30 mV, equal both for AlPcS_2_ and AlPcS_4_.

One of the simplest consequences of the model is that the ratio of R_c_ and R_t_ is always less than 1. This ratio is equal to

T5 $$\frac{{R}_{c}}{{R}_{t}}=\frac{{b}_{1}}{{b}_{2}+\alpha {S}_{0}}=\frac{{k}_{c-t,m}+{k}_{W}}{{k}_{c-t,m}+{k}_{W}+{k}_{S,O}({T}_{0}+{S}_{0})}$$

## Discussion

Our experiments revealed that SO in membranes is extremely short-lived: instead of spending microseconds in the membrane interior, SO does not survive tens of nanoseconds. In other words, *τ*_r_ ≈ *τ*_l_. *τ*_l_ is mainly governed by the time it takes SO to reach its nearest target. The conclusion is based on experiments, in which a SO molecule that was born in the membrane headgroup region was free to target molecules in (i) the same headgroup region, (ii) the inner hydrophobic layer, and (iii) the opposing headgroup region. Although the increment in distance between these targets was smaller than 2 nm, the probability of SO reacting with the targets sharply decreased with distance.

A lower limit of *τ*_l_ maybe obtained by assuming a diffusion limited reaction. In our experiments, the DT molecules (for *φ*_ads_ = 20 mV) were only 4 nm apart as indicated by FCS measurements. Thus, in *cis* configuration, the distance *r* between a PS and the nearest DT neighbor molecule did not exceed 2 nm. Taking into account SO’s diffusion coefficient *D* = 5 × 10^−5^ cm^2^ s^−1^ ^7^, we find the SO travel time

6 $${\tau }_{t}=\frac{{r}^{2}}{D}=0.8\,ns$$

*τ*_t_ is probably smaller in biological membranes since they are densely packed with SO scavengers that are represented by unsaturated lipids and membrane proteins containing aromatic residues^30–33^.

In order to obtain a more accurate estimate of *τ*_l_, we performed a quantitative analysis of our experiments by solving the differential equations for the combined system of chemical reactions and diffusion processes (see Theory). We found the simplified solution represented by Eq. (7):

7 $$\frac{{R}_{t}}{{R}_{c}}=1+\frac{{k}_{S,O}({T}_{0}+{S}_{0})}{{k}_{c-t,m}+{k}_{W}}$$

where *T*_0_, *S*_*0*_, *R*_*c*_, and *R*_*t*_ denote *T* prior to illumination, the initial surface density of DT’s oxidizable moiety that does not contribute to *Δφ*_*b*_ (Fig. 6), *R* in *cis* and *trans* configurations, respectively. The *R*_*t*_/*R*_*c*_ ratio depends on the combined rate *k*_*W*_ of SO membrane exit and quenching by the aqueous medium, the transfer rate *k*_*c*-*t*,*m*_ from the outer membrane layer to the medium layer, and the rate *k*_*S*,*O*_ of SO quenching by the *S* moiety of DT (Fig. 6). Eq. 7 assumes a non-negligible value *S*_0_, i.e. it does not work for *T*_0_ ≫ *S*_0_. Equation (7) predicts that *R*_*c*_ is always smaller than *R*_*t*_, which is in perfect agreement with the experiment.

We first globally fitted the complete set of differential equations (see Theory) to individual sets of *R*_c_ = f(T_0_) and *R*_t_ = f(T_0_) that have been experimentally obtained for both AlPcS_4_ and AlPcS_2_ (Table 1 and Fig. 5A,B) and then constructed the R_t_/R_c_ = f(*T*_0_) plot from the results of the fit (Fig. 5C). Solving the complete set of equations also allowed us to obtain the rate *k*_Gen_ of SO generation. *k*_Gen_ is fourfold higher for AlPcS_2_ than for AlPcS_4_, indicating either an environmental effect on SO yield or immediate SO escape into the aqueous solution without ever entering the lipid membrane. Indeed, in contrast to AlPcS_4_’s four negative charges, which ensure that it lies flat on the membrane surface, AlPcS_2_ penetrates into the bilayer. Its chromophore ring inclines towards the hydrocarbon core as indicated by a substantial contribution of *Δφ*_d_ to *Δφ*_b_. Thus, AlPcS_2_’s ring is exposed to a four-fold higher oxygen concentration than that belonging to AlPcS_4_. The observed increment in *k*_Gen_ agrees well with the previously reported increase of photosensitizer efficiency with increasing hydrophobicity^11,34,35^.

Our mathematical model revealed the reaction rates of SO with targets: The rate *k*_*T*,*O*_ of SO quenching by the *T* moiety of DT is roughly equal to k_S,O_ ≥ 6.0·10^−10^ m^2^s^−1^. Taking into account the target density of about 0.1 molecule nm^−2^, this value translates into a reaction time *τ*_T,O_ ≈ 16 ns of the *T* moiety with SO. *τ*_T,O_ is three orders of magnitude smaller than the estimate for *τ*_l_ from experiments^36^ with non-oxidizable lipids, underpinning the conclusion that the main determinant of SO lifetime is target density. Moreover, *τ*_l_ is not diffusion limited, i.e. it is not governed by *τ*_t_: *τ*_l_ ≈ *τ*_T,O_ ≫ *τ*_t_.

The mathematical model also allows assessing *τ*_*dw*_. From *k*_*m*,*c*−*t*_ = 5.5 × 10^7^ s^−1^ and *k*_*w*_ = (5.0 ± 0.9) × 10^7^ s^−1^ (Table 1) we find *τ*_*dw*_ ~ (*1/k*_*m*,*c*−*t*_) + (*1/k*_*w*_) ≈ (40 ± 8) ns. This estimate agrees reasonably very well with the one derived from the literature values of SO’s distribution coefficient and diffusion constant (see Introduction). *τ*_l_ < *τ*_dw_ indicates that most of the reactive oxygen species stay in the membrane long enough to react with the target, i.e. that *τ*_r_ is determined by *τ*_l_. However, the values of *τ*_l_ and *τ*_dw_ are too close to each other to ensure a photodynamic efficiency of 100%. An increase in targeting efficiency can be realized by two different approaches: (i) by reducing the separation between PS and target so that other molecules susceptible to SO, like unsaturated lipids, cannot protect the proteinaceous target from encounters with SO and (ii) by increasing the quantum yield of PS molecules. Our model allows determining *k*_Gen_ in a membrane environment, thereby offering an important tool for PS optimization.

## Electronic supplementary material

Supplementary Information

## Data Availability

The datasets generated during and/or analyzed during the current study are available from the corresponding author on reasonable request.
