# Supplementary material for: Residence time of singlet oxygen in membranes
Source: Sci Rep. 2018 Sep 18;8:14000. doi: 10.1038/s41598-018-31901-9 (PMC6143606; doi:10.1038/s41598-018-31901-9)
Supplement: Supplementary file 1 — Supplementary Information [file 41598_2018_31901_MOESM1_ESM.pdf]

Supplementary information

To:

Residence time of singlet oxygen in membranes

By:

V.S. Sokolov, O.V. Batishchev, S.A. Akimov, T.R. Galimzyanov, A.N. Konstantinova, E. Malingriaux, Y.G. Gorbunova, D.G. Knyazev, P. Pohl

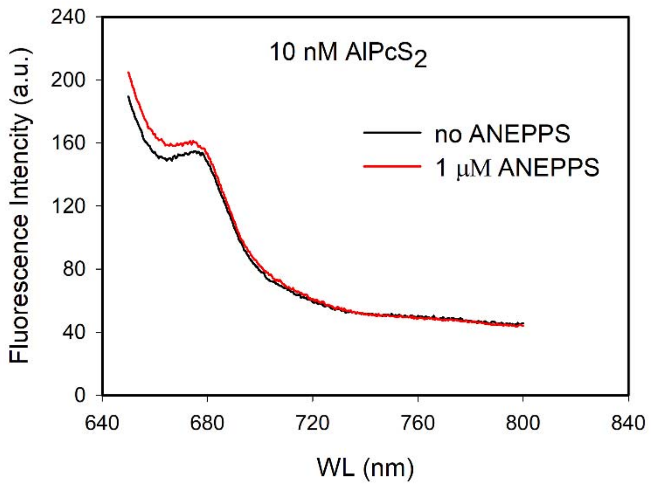

Fig. S1 The spectra of fluorescence of 10 nM $AlPcS_2$ in the absence (black curve) and presence (red curve) of 1 μM of di-4-ANEPPS. Excitation was at 604 nm. The water solution was the same as in the legend to Fig. 1. The vesicles were prepared from diphytanoylphosphocholine by extrusion through polycarbonate membranes with 100 nm pores. Final concentration of lipid in water solution was 1 mg/ml.
